# Impacts of Climate Change on Health and Wellbeing in South Africa

**DOI:** 10.3390/ijerph15091884

**Published:** 2018-08-31

**Authors:** Matthew F. Chersich, Caradee Y. Wright, Francois Venter, Helen Rees, Fiona Scorgie, Barend Erasmus

**Affiliations:** 1 Wits Reproductive Health and HIV Institute, Faculty of Health Sciences, University of the Witwatersrand, Johannesburg 2000, South Africa; FVenter@wrhi.ac.za (F.V.); hrees@wrhi.ac.za (H.R.); FScorgie@wrhi.ac.za (F.S.); 2 Environment and Health Research Unit, South African Medical Research Council and Department of Geography, Geoinformatics and Meteorology, University of Pretoria, Pretoria, Hatfield, Private Bag X200028, South Africa; Caradee.Wright@mrc.ac.za; 3 Global Change Institute, University of the Witwatersrand, Johannesburg 2000, South Africa; Barend.Erasmus@wits.ac.za

**Keywords:** South Africa, climate change, HIV, eco-migration, extreme weather events, health

## Abstract

Given its associated burden of disease, climate change in South Africa could be reframed as predominately a health issue, one necessitating an urgent health-sector response. The growing impact of climate change has major implications for South Africa, especially for the numerous vulnerable groups in the country. We systematically reviewed the literature by searching PubMed and Web of Science. Of the 820 papers screened, 34 were identified that assessed the impacts of climate change on health in the country. Most papers covered effects of heat on health or on infectious diseases (20/34; 59%). We found that extreme weather events are the most noticeable effects to date, especially droughts in the Western Cape, but rises in vector-borne diseases are gaining prominence. Climate aberration is also linked in myriad ways with outbreaks of food and waterborne diseases, and possibly with the recent Listeria epidemic. The potential impacts of climate change on mental health may compound the multiple social stressors that already beset the populace. Climate change heightens the pre-existing vulnerabilities of women, fishing communities, rural subsistence farmers and those living in informal settlements. Further gender disparities, eco-migration and social disruptions may undermine the prevention—but also treatment—of HIV. Our findings suggest that focused research and effective use of surveillance data are required to monitor climate change’s impacts; traditional strengths of the country’s health sector. The health sector, hitherto a fringe player, should assume a greater leadership role in promoting policies that protect the public’s health, address inequities and advance the country’s commitments to climate change accords.

## 1. Introduction

The question of how to tackle the ecological determinants of health is poised to become the signature public health issue of the coming decade in South Africa, in much the same way that HIV took centre stage in the preceding decades. Rapid environmental changes are creating observable effects in multiple domains, from air quality, temperature and weather patterns, to food security and disease burden [1]. Ambient air pollution is estimated to have been responsible for 4% of deaths in South Africa in 2015 [2]. Even though there is some spatial variation in the warming signal, most of South Africa has experienced upward trends in temperature during the last half of the 20th century [3,4]. Food security is also under threat, with, for example, crop yields likely to decline in many parts of the country, accompanied by livestock losses [5]. Furthermore, it is now possible to detect changes in response to climate change in most terrestrial, freshwater and marine ecosystems in the country [6,7,8]. Species are changing genetically, physiologically and morphologically, and their distribution is shifting, which affects food webs, and foments transmission of infectious diseases [9].

In South Africa, despite policies promoting an ambitious renewable energy programme, the country’s response to climate change has been hampered by policy uncertainty and corruption, especially in the energy and transport sectors, and its health systems are ill-prepared for the effects of climate aberration [10]. The new National Climate Change Bill, which is currently open for public comment shows promise, however. Its provisions for coordination among different government departments have the potential to remove policy uncertainty, and align related policies [11].

This article summarises evidence of the impact of climate change on health in South Africa and highlights specific effects on vulnerable populations. The review covers the direct impacts on health through extreme events and temperature increases, but also the more indirect impacts mediated through natural systems (for example infectious diseases) and through social vulnerabilities [12]. While several narrative reviews have summed the impacts of climate change on health in South Africa [13,14,15,16], there is a need to review more up to date literature and to collate evidence in a systematic manner.

## 2. Review Methods

**Figure 1:**
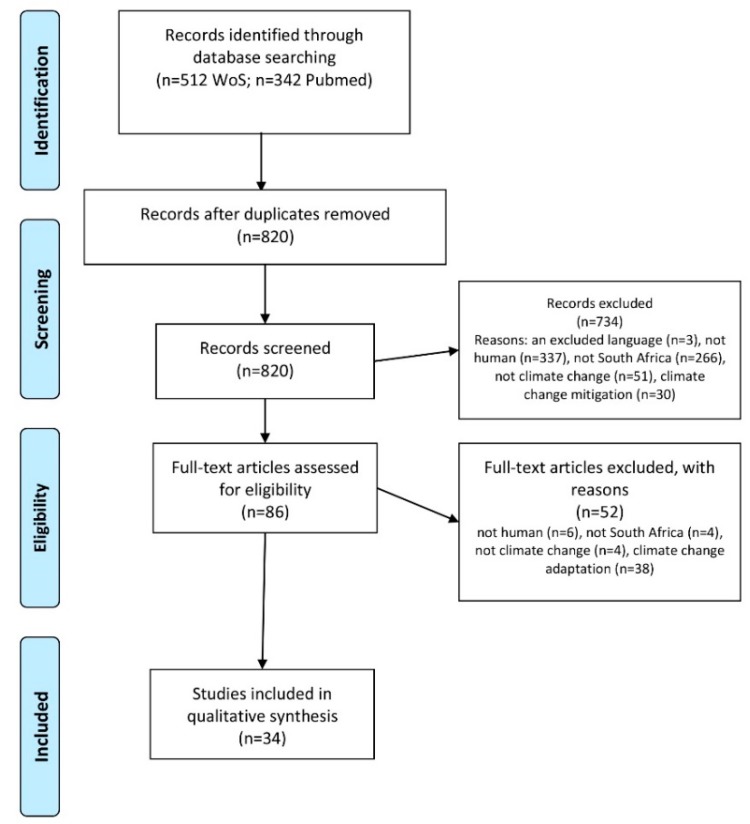
PRISMA Flow diagram for review of articles on impact of climate change in South Africa [17].

Medline (PubMed) and Web of Science were searched on 8 August 2018 (Supplementary File 1: review protocol). The PubMed search strategy included free text terms and MeSH codes, specifically: (((((“South Africa”[MeSH]) OR (“South Africa”[Title/Abstract]) OR (“Southern Africa*“[Title/Abstract]))) AND “last 10 years”[PDat])) AND (((“global warming”[Title/Abstract] OR “global warming”[MeSH] OR climatic*[Title/Abstract] OR “climate change”[Title/Abstract] OR “climate change”[MeSH] OR “Desert Climate”[MeSH] OR “El Nino-Southern Oscillation”[MeSH] OR Microclimate[MeSH] OR “Tropical Climate”[MeSH])). A total of 820 titles and abstracts were screened by a single reviewer after removal of 34 duplicate items (Figure 1).

To be included, articles had to describe the impact of climate change on health in South Africa. All study designs were eligible, including modelling studies, narrative and systematic reviews, case studies, case series and qualitative research. We excluded articles that were not in English (*n* = 3), only covered animals or plants (*n* = 343), were not on South Africa (*n* = 270), were unrelated to health (*n* = 47) or climate change (*n* = 55), or were on climate change adaptation (*n* = 38) or mitigation (*n* = 30). We then screened full text articles for eligibility (*n* = 86), of which 34 were included. We then extracted data on the characteristics of the included articles (Table 1). The findings presented in each included article were used to draft the text of the review. We also included additional articles located through searches of article references or through targeted internet searches for policy documents, for example.

## 3. Results

A quarter of the articles included South Africa and another country (8/34; 24%; Table 1). Fully 41% of the papers were narrative reviews or editorials (14/34). A further 29% were modelling studies (10/34). Only one article was located that applied qualitative methods. One quarter addressed the effects of heat on health in the country (9/34; 26%), while a third investigated the impact of climate change on infectious diseases (11/34; 32%). Six of these 11 papers addressed malaria. Below, we present a summary of the key findings from the literature reviewed.

### 3.1. Direct Effects of Temperature Rises and Eextreme Weather

Observed rates and modelled projections indicate that warming over southern Africa is happening at twice the global rate [48]. Unless concerted international action is taken to reduce greenhouse gas emissions, temperatures may rise more than 4 °C over the southern African interior by 2100, with increases of more than 6 °C over large the western, central and northern parts of the country, which have faced several years of droughts [30,49]. Relative to a 1981–2000 base period, the probability of summer heat waves over South Africa has increased by over 3.5 fold [50]. Impacts of heat waves differ from the more insidious, but no less harmful, rises in heat that increasingly cause heat-related symptoms during Summer, but also in Spring and Autumn in some parts of the country [30].

An analysis using national mortality and temperature data for each district of the country over 17 years found that temperature-related mortality (from cold or hot spells) accounts for 3.4% of deaths in South Africa [51]. Those at extremes of age are most vulnerable, given their reduced thermoregulatory ability, as well as more limited mobility and resources to adjust to extreme temperatures. A study pooling data from Cape Town, Durban and Johannesburg, calculated that for every 1 °C rise, overall mortality escalates by 1% and by 2% in those aged above 65 years [52]. Heat exposures also hold particular dangers for pregnant women and the developing foetus, a concern given the already high levels of maternal and infant mortality in the country [53,54].

As temperatures rise, the levels of risk from occupational heat may increase from ‘low risk’ to ‘moderate or high risk’, especially in the mining, agriculture and outdoor service sectors [32]. A seminal study in the mid-20th century of > 200,000 underground miners in South Africa reported a mortality rate of 3.3 deaths/year/1000 miners if the temperature exceeded 34 °C, compared to 0.7 deaths/year/1000 miners when temperatures were between 31 and 33 °C [55]. Outdoor workers in Upington, one of the hottest part of the country, frequently experience heat-related effects, including sunburn, sleeplessness, exhaustion and reduced productivity [36]. At the time of the study, few, if any, measures had been taken to reduce these effects.

Impacts of heat in the domestic environment are also important to consider [56]. Low-cost government-built housing in South Africa and informal settlement houses (mostly made of sheets of corrugated iron, bricks, wood and plastic) are poorly insulated against heat and cold. During hot weather these structures may be 4–5 °C warmer than outdoor temperatures and cooler during cold spells by the same magnitude [39,40,57]. Replacing informal settlement housing with formal brick and cement housing could reduce heat-related mortality by as much as a half [40]. Similarly, many school classrooms in the country are constructed of prefabricated asbestos sheeting with corrugated iron roofing, are overcrowded and lack ceiling fans [23]. Temperatures in these structures often exceed 30 °C and heat-health related symptoms are commonplace [23]. Equally concerning is the evidence that temperatures in many waiting rooms in public-sector health facilities are dangerously high. A study of eight rural clinics found that the temperature in these clinics was as much as 4 °C higher than outdoors, reaching temperature ranges associated with heat-health impact warning categories of ‘caution’ and ‘extreme caution’ [47]. In addition, already vulnerable inner-city areas, such as Hillbrow, Johannesburg [58] constitute urban ‘heat islands’, where temperatures can exceed that of sub-urban areas by several degrees [59].

Climate change is also projected to increase the frequency and severity of storms and flooding in the country [60]. The effects of these extend beyond mortality alone, and include injuries, food and water insecurity, spread of disease and mental health conditions [61].

### 3.2. Indirect Effects of Climate Change on Infectious Diseases

Temperature increases have sizable implications for the transmission of vector-borne diseases. Rises in temperature as well as precipitation changes have been linked with malaria spikes, especially in Limpopo province [21,33]. These spikes often occur around two months after warmer temperatures and high precipitation have occurred in neighboring countries [28]. The projected changes in climate in South Africa will favor the survival of the malaria vector *Anopheles arabiensis*, while its distribution may decline in many other parts of Africa [19,25,43]. The effects of climate variability are especially notable with Rift Valley Fever, which mostly affects the semi-desert Karoo biomes during strong La Nina years, but then during El Nino episodes shifts to the central grassland areas of South Africa [52,62]. Changes in the distribution of the disease vectors *Aedes aegypti* and *Aedes albopictus* have raised the likelihood of transmission of dengue fever, Zika and other infections in the region [61]. The Avian influenza epidemic in South Africa in 2017, attributed, in part, to climate change, [63] threatens poultry food sources, among other things. Warming may also alter the distribution, breeding and survival of the snail species implicated in schistosomiasis, whose choice of habitat is highly sensitive to water temperature [29].

Importantly, climate change impacts on the persistence and dispersal of water- and food-borne pathogens in a myriad of ways. Droughts and high precipitation worsen water quality, and hamper hand washing and other hygiene practices [64,65]. Bacterial pathogens multiply faster in higher temperatures, which also can result in breakdowns in food cooling chains. A study in children under-five in Cape Town noted that a 5 °C rise in minimum weekly temperatures increased cases of diarrhoea by 40% one week thereafter [37]. These findings echo a similar study in Limpopo [42]. An escalation in sea-surface temperature was linked to a cholera outbreak in 2000–2001 in KwaZulu-Natal, possibly stemming from an increased abundance of phytoplankton and thus higher numbers of copepods (small crustaceans commensal to cholera) that feed on these organisms [66].

Listeria monocytogenes, recently responsible for a major epidemic across South Africa, appears to be particularly climate sensitive, with occurrences rising with temperature spikes [24,67,68]. In food processing plants, water scarcity hampers efforts to clean food-processing machines. Scarcity may also shift the sources of water used for agriculture and domestic purposes, raising exposure to *Listeria* and other pathogens in South Africa [27,69].

### 3.3. Indirect Effects of Climate Change on Mental Health

The mental health impacts of extreme weather may compound the multiple health and social stressors that already beset many South Africans. The country has amongst the highest levels of mental illness worldwide, much of which is linked to high levels of gender-based and other forms of violence, crime, poverty, inequality, HIV and political turmoil [70,71]. Climate change could possibly signal a tipping point for many citizens. Though this issue is of major importance, no studies in South Africa were located in our review that directly address interactions between climate change and mental health. It is especially important to determine if risky sexual behaviours in South Africa increase following disasters or migration related to climate change. This outcome is plausible given that risky behaviours, in general, tend to rise in these circumstances [72]. Some newspaper reports have suggested that the recent drought in the country is associated with elevated suicide rates among farmers, but additional research is needed on this question [73,74].

### 3.4. Effects of Climate Change on Specific Population Groups

It is one of the tragic ironies of our times that the population groups that have contributed least to greenhouse-gas emissions are the first and the hardest hit by the effects of these emissions. South Africa is ranked as the second most inequitable country in the world, where the poorest 20% of the population consumes less than 3% of total expenditure, and the wealthiest 20% consumes 65% [75].

Our ability to adapt to climate change may be shaped by the same inequalities that have become the fault lines of society in South Africa, above all, gender [35]. Rates of gender-based violence in the country are amongst the highest in the world, affecting 1 in 3 women during their lifetime [76]. Again, it would be important to investigate whether these rates rise even further following extreme weather events in the country, as has been reported elsewhere [77]. During food insecurity in the country, women and girls disproportionately suffer health consequences of nutritional deficiencies and carry additional burdens, such as travelling further to collect water during droughts [35]. According to a UNICEF report, children in South Africa are especially vulnerable to climate change, facing higher risks during extreme weather, malnutrition during food shortages, and respiratory disease from increases in pollen and dust [78]. Of the 57 million people in South Africa, about 8 million are infected with HIV, the largest number worldwide [54,79]. Immunocompromised populations may also be more susceptible to the increased pathogen loads associated with higher temperatures, when factors such as stigma and poor health have already undermined their resilience. Migration, which is related in part to climate change, creates additional risks for HIV acquisition [80]. Clearly, the interface between climate change and HIV in South Africa is complex and warrants careful study [18].

Extreme weather conditions will have considerable impacts on those who depend on climate-sensitive resources and ecosystems for their livelihoods. Soil drying, coupled with irregular rainfall caused by rising temperatures, particularly in the western regions of South Africa [20], constrains agricultural production. This is potentially most harmful for subsistence farming communities who are already prone to geopolitical and economic marginalization. Rainfall shortages, higher temperatures and reduced soil moisture have all been associated with internal migration in the country [81]. This is especially true of communities that are highly dependent on natural resources for their livelihood (e.g., firewood, seeds and wild foods) [34]. Movement to vulnerable inner-city areas and informal settlements is particularly concerning as these areas have the highest risks of HIV transmission [18,58,82]. These patterns, termed eco-migration, can accentuate the already high levels of urbanization in the country. Of note, migration carries increased risks of sexual violence for women and some female migrants in South Africa may have few options for survival other than sex work [83]. Eco-migration also erodes social networks—the practical and emotional resources underpinning health and well-being—and has fomented conflict in sub-Saharan Africa [26]. The potential for this to happen also in South Africa is high, as migration related to climate change may bring already festering xenophobic attitudes to the fore, and generate conflict and violence [26].

## 4. Conclusions

When climate change is framed as a predominately a health issue, rather than purely as an environmental, economic, or technological challenge, it becomes clear that South Africa is facing major challenges. Health puts a human face on what can sometimes seem to be a distant threat [84]. Through a deeper engagement with the topic, health professionals, hitherto fringe players, could lead the way in identifying impacts of climate change, addressing these and pushing for mitigation against further deterioration. Without these interventions, climate change will likely worsen the country’s existing socioeconomic and health inequities [22]. A step-change is required in the country’s response if it is to meet its commitments to the Sustainable Development Goal 2, which includes improved food security and improved nutrition, and sustainable agriculture [5].

It is significant that very few studies were identified in our review used empirical data from health services to analyze the impact of climate change. More effective use of surveillance and research data are required to monitor climate change impacts on human health in South Africa, and to then bring these findings to the attention of policy makers and the public. The considerable human resources dedicated to health research in the country have yet to turn their attention to climate change [38]. Equally, many of the leaders in climate change research globally are South African, but they have yet to focus on climate change and health [15]. A few carefully targeted funding opportunities may help to induce these shifts (Box 1), especially if driven by a proactive inter-sectoral research agenda applying a full spectrum of research methodology, from modelling to social science enquiry. Notably, the review included only one qualitative research paper [31]. Perhaps most importantly, additional detection and attribution studies are needed in South Africa, documenting the extent to which changes in health can be attributed to climate change [51,85]. These studies can inform risk management and planning for future changes, but require reliable long-term data sets, and more knowledge about the factors that confound and modify the effects of climate on health [86].

Box 1 **Research agenda for Climate Change and Health in South Africa.**

***Key Research* *Priorities*:**

1. Examine the interface between climate change and HIV, identifying possibilities for a joined-up, synergistic, evidence-based response to climate-HIV interactions.

2. Attribution and detection studies that use long-term, multi-decadal climate data to document and project long-term trends in health outcomes. Most such work has focused on malaria, with substantial gaps in data on diarrheal diseases, for example. Predicting other emerging infectious diseases with more complex environmental determinants remains a significant challenge.

3. Document the heat impacts on vulnerable groups, such as infants and the elderly, and interactions between heat and poor air quality in occupational and domestic settings.

4. Understand heat exposure in occupational settings in South Africa and develop interventions to reduce health-health, especially for miners, agricultural workers and those providing outdoor services.

5. Investigate the effects of climate change on mental health, especially among vulnerable population groups.

6. Identify the indirect impacts of a changing climate on food security and other social determinants of health.

Importantly, the measurement and communication of the impact of climate change could be improved by drawing on the considerable policy, research, advocacy and communication expertise built up during the HIV response in South Africa. By becoming proficient communicators on the subject of climate change, health professionals could provide clear messaging on risks and actions required during extreme weather, for example. Just as critical is the need for health professionals to use their considerable influence to advocate for policy change and improved climate governance. The overarching priority ultimately is to enact policies to shift the country away from a coal-dependent energy system and economy, and to encourage the public to question its seemingly boundless desire for economic growth and consumer goods. This requires honest reflection on whether South Africans can ‘develop the enhanced moral imagination to motivate doing better and more with less, in time to effect meaningful change’ [87]. It is time for the health sector to position itself at the core of such changes.

## Figures and Tables

**Table 1:** Characteristics of studies included in the review.

No	Article Title	Country(ies) of Research	Study Setting	Study Design	Study Population	Study Aim	Study Outcome Data
1	The HIV/AIDS epidemic in South Africa: Convergence with tuberculosis, socio-ecological vulnerability, and climate change patterns [18]	South Africa	Whole country	Review	Persons living with HIV or TB, as well as the general population	Review intersections between HIV, tuberculosis and climate change	N/A
2	Modelling the influence of temperature and rainfall on the population dynamics of Anopheles arabiensis [19]	South Africa	Dondotha village, northeast part of KwaZulu-Natal Province	Modelling study	Anopheles arabiensis mosquitos	To apply a climate-based, ordinary-differential-equation model to analyse the influence of ambient temperature on the development and the mortality rate of Anopheles arabiensis	The seasonality of An. arabiensis and the influence of climatic factors on the vector population dynamics
3	Potential impacts of climate change on extreme precipitation over four African coastal cities [20]	Egypt, Mozambique, Nigeria, South Africa	Cape Town, Western Cape	Modelling study	General population living in the four cities	To examine the impacts of climate change on extreme precipitation events under different climate scenarios	Number of extreme precipitation events, number of wet days and dry spells
4	Climatic Variables and Malaria Morbidity in Mutale Local Municipality, South Africa: A 19-Year Data Analysis [21]	South Africa	Mutale municipality, Limpopo province	Time-series analysis	Patients with malaria	To examine the dynamics of the disease’s transmission and its persistence, by investigating the relationship between climate and the occurrence of malaria	Monthly climatic variables and monthly malaria cases using data over 19 years. Time lag between climate variation and malaria incidence
5	Why equity in health and in access to health care are elusive: Insights from Canada and South Africa [22]	Canada, South Africa	Whole country	Narrative review	Whole population, but focus on vulnerable groups	To illustrate the complexity of achieving greater equity, drawing on experiences in Canada and South Africa. Also to Identify bi-directional lessons relevant both to countries and globally concerning health care funding approaches and other means of reducing health inequities, including those related to climate change	N/A
6	Students' Perceived Heat-Health Symptoms Increased with Warmer Classroom Temperatures [23]	South Africa	City of Joburg, Gauteng Province	Cross-sectional study	School children aged 14–18 years at 8 schools in City of Johannesburg	To assess school children‘s perceived heat-health symptoms during school hours in the classroom	Self-completed heat-health log and questionnaire, and indoor temperature and relative humidity measured in classrooms
7	How climate change can fuel listeriosis outbreaks in South Africa [24]	South Africa	Whole country	Editorial	General population	To consider the relationship between listeriosis outbreaks and climate change	N/A
8	Ecological niche and potential distribution of Anopheles arabiensis in Africa in 2050 [25]	All of Africa	Whole country	Modelling study	Anopheles arabiensis mosquitos	To map the future potential distribution of Anopheles arabiensis in Africa	Distribution of Anopheles arabiensis under three climate change scenarios, comparing baseline and projected changes
9	Environmental Change, Migration, and Conflict in Africa: A Critical Examination of the Interconnections [26]	All of sub-Saharan Africa	Migrants in the country	Narrative review	People who are migrants, or in conflict areas	To examine interconnections between environmental change, migration and conflict in Africa, analysing evidence for migration as an intermediary and bidirectional causal variable in the climate-conflict interaction	N/A
10	The health implications of wastewater reuse in vegetable irrigation: a case study from Malamulele, South Africa [27]	South Africa	Malamulele, Limpopo province	Cross-sectional survey	Farmers, children, farm workers, vegetable consumers and environmental samples	To investigate the health and socio-economic implications of irrigation of vegetables with wastewater	Number and type of helminth eggs in wastewater and vegetable wash water, prevalence of gastroenteritis
11	Seasonally lagged effects of climatic factors on malaria incidence in South Africa [28]	South Africa	Limpopo	Spatial and temporal mapping, with self-organizing maps	Malaria cases	To analyse the relationship between local climatic effects and remote atmospheric teleconnections on the incidence of malaria, including lag effects	Association between malaria incidence and local and regional climate factors
12	Effect of temperature on the Bulinus globosus—Schistosoma haematobium system [29]	South Africa	uMkhanyakude and Verulam, KwaZulu-Natal Province	Prospective study	Bulinus globosus and Schistosoma haematobium	To assess the effect of temperature on snail fecundity, growth, survival and parasite development	Snail fecundity and growth., and parasite development
13	Current and Potential Future Seasonal Trends of Indoor Dwelling Temperature and Likely Health Risks in Rural Southern Africa [30]	South Africa	Giyani, Limpopo Province	Cross-sectional study	Households in rural setting in north-eastern part of South Africa	To consider the relationship between temperatures in indoor and outdoor environments in a rural residential setting in a current climate and warmer predicted future climate	Temperature and humidity measurements collected hourly in 406 homes in summer and spring and at two-hour intervals in 98 homes in winter
14	A socio-economic approach to One Health policy research in southern Africa [31]	All countries in Southern Africa	Whole country	Narrative review	General population	To identify which factors affect the burden of disease and how the burden could affect socio-economic well-being, including climate change	N/A
15	Climate change and occupational health: A South African perspective [32]	South Africa	Whole country	Narrative review	People in the workplace	To review the impacts of climate change on occupational health and possible prevention and control measures	N/A
16	Long-run relative importance of temperature as the main driver to malaria transmission in Limpopo Province, South Africa: a simple econometric approach [33]	South Africa	Limpopo Province	Econometrics	People with malaria	To examine the distribution of malaria, determine direction and strength of the relationship and causality between malaria and meteorological variables	Malaria correlation with temperature and rainfall
17	Spatially and Temporally Varying Associations between Temporary Outmigration and Natural Resource Availability in Resource-Dependent Rural Communities in South Africa: A Modeling Framework [34]	South Africa	Rural areas	Modelling study	Agincourt, rural area of Mpumalanga province	To methodologically assess the robustness of migration environment associations and to explore the effects of inherent spatial variation of these associations	Spatial variability in migration-environment associations. Indicators of natural resource availability.
18	Mind the gap: institutional considerations for gender-inclusive climate change policy in Sub-Saharan Africa [35]	All of sub-Saharan Africa	Whole country	Narrative review	General population, vulnerable groups of women	To elucidate why women should be placed at the heart of climate change interventions and establish connections between gender and climate change	N/A
19	Climate change impacts on working people (the HOTHAPS initiative): findings of the South African pilot study [36]	South Africa	Johannesburg, Gauteng Province and Upington, Northern Province	Qualitative study	People working in sun-exposed environment, including grave diggers, street sweepers, roadside construction workers, sewage and sanitary workers and horticultural workers.	To investigate the perceptions of outdoor workers regarding their work environment in hot weather and how this affected their health and productivity	Heat-related effects, including sunburn, sleeplessness, irritability, and exhaustion. Also work levels, outputs and adaptation measures
20	Climate change: A threat towards achieving ‘Sustainable Development Goal number two’ (end hunger, achieve food security and improved nutrition and promote sustainable agriculture) in South Africa [5]	South Africa	Whole country	Narrative review using snowball sampling to select sources and discourse analysis	General population	To examine the impacts of climate change on the achievement of SDG 2	N/A
21	Temperature Variability and Occurrence of Diarrhoea in Children under Five-Years-Old in Cape Town Metropolitan Sub-Districts [37]	South Africa	Cape Town, Western Cape	Surveillance data longitudinal analysis	Children under five with diarrhoea	To describe the relationship between temperature change and diarrhoea in under five-year-old children	Incident cases of diarrhoea and associations with temperature
22	Responding to climate change in southern Africa—the role of research [38]	Southern Africa	Whole country	Editorial	Academic researchers and funders	To highlight need for collecting locally relevant information on climate change	N/A
23	A public health approach to the impact of climate change on health in southern Africa—identifying priority modifiable risks [14]	Southern Africa	Whole country	Editorial	General population	To describe a conceptual model for analysing climate-related health risks ranging from distal and infrastructural, to proximal and behavioural, and their relation to the burden of disease	N/A
24	Indoor Temperatures in Low Cost Housing in Johannesburg, South Africa [39]	South Africa	City of Johannesburg, Gauteng Province	Cross-sectional study	Five impoverished suburbs in City of Johannesburg	To record indoor temperature and relative humidity in homes	For 100 homes, collected indoor temperature and relative humidity as well as ambient data for the suburb
25	The impact of housing type on temperature-related mortality in South Africa, 1996–2015 [40]	South Africa	Eastern and Western Cape Provinces	Modelling study	People living in five types of housing	To examine how housing modifies temperature-mortality associations	Temperature-related mortality burdens
26	Potential impacts of climate change on wildfire dynamics in the midlands of KwaZulu-Natal, South Africa [41]	South Africa	Midlands area of KwaZulu Natal Province	Modelling study	People and vegetation in study area	To investigate fire dynamics under different climatic scenarios	Annual average fire danger
27	Climate change is catchy-but when will it really hurt? [16]	South Africa	Whole country	Editorial	General population with a focus on impacts on vulnerable groups	To review and discuss the possible impacts of climate change on health and call for more climate-health research	N/A
28	Impact of climate change on children's health in Limpopo Province, South Africa [42]	South Africa	5 municipalities in Limpopo Province	Surveillance data longitudinal analysis	Children under 13 attending a hospital	To examine the impact of climate change on child health, including trends and urban-rural variation in disease, and to suggest adaptation/mitigation strategies.	Association of temperature and rainfall with disease incidence
29	Zoom in at African country level: potential climate induced changes in areas of suitability for survival of malaria vectors [43]	Africa	Whole country	Modelling study	Anopheles arabiensis and Anopheles gambiae mosquitos	To estimate the geographical distribution and seasonal abundance of malaria vectors in relation to climate factors	Survivorship of malaria vectors. Change in malaria suitability zones
30	Heat effects of ambient apparent temperature on all-cause mortality in Cape Town, Durban and Johannesburg, South Africa: 2006–2010 [44]	South Africa	Cape Town, Western Cape Province; Durban, Kwa-Zulu Natal Province; Johannesburg, Gauteng Province	Modelling study	People who died	To investigate associations between daily ambient apparent temperature and daily all-cause non-accidental mortality	Death rates at different temperatures
31	Human health impacts in a changing South African climate [45]	South Africa	Whole country	Narrative review	General population with a focus on impacts on vulnerable groups	To consider impacts of climate change on human health and suggest ways to prevent adverse impacts	N/A
32	Climate change: One of the greatest threats to public health in the 21st century [46]	South African	Whole country	Editorial	General population	To consider impacts of climate change on human health	N/A
33	Indoor Temperatures in Patient Waiting Rooms in Eight Rural Primary Health Care Centers in Northern South Africa and the Related Potential Risks to Human Health and Wellbeing [47]	South Africa	Giyani, Limpopo Province	Cross-sectional study	Eight clinic waiting rooms	To determine indoor temperatures of waiting rooms in eight rural primary health care facilities during a 6-month period.	10-minute temperature and relative humidity readings that were used to calculate apparent temperature (real-feel)
34	Climate change impacts and adaptation in South Africa [15]	South Africa	Whole country	Narrative review	General population	To review current approaches and recent advances in research on climate impacts and adaptation	N/A

N/A: not applicable.

## Supplementary Materials

The following are available online at http://www.mdpi.com/1660-4601/15/9/1884/s1, File 1, Protocol for systematic review of Climate Change research in South Africa.
