# Supplementary material for: Impacts of Climate Change on Health and Wellbeing in South Africa
Source: Int J Environ Res Public Health. 2018 Aug 31;15(9):1884. doi: 10.3390/ijerph15091884 (PMC6164733; doi:10.3390/ijerph15091884)
Supplement: Supplementary file 1 [file ijerph-15-01884-s001.pdf]

International Journal of
*Environmental Research
and Public Health*

MDPI

Supplementary

Impacts of Climate Change on Health and Wellbeing in South Africa

Matthew F. Chersich, Caradee Y. Wright, Francois Venter, Helen Rees, Fiona Scorgie and Barend Erasmus [3]

Protocol for systematic review of Climate Change research in South Africa

Summary of the review process

The review screening and data extraction has 4 stages, namely:

- **Stage 1:** Design search strategy and upload records into Endnote
- **Stage 2:** Screening of titles and abstracts for eligibility
- **Stage 3:** Review of screen positive full text articles, and screening of full text articles for eligibility
- **Stage 4:** Extracting data from full text articles

The process of the review follows the PRISMA flow chart and guidelines.

Inclusion and exclusion criteria for the review

Inclusion criteria:

1. Articles that describe the impact of climate change on South Africa.
2. Studies set in South Africa, though may be part of multi-country studies, such as those involving Southern African countries. Studies that address 'Southern Africa' as a broad topic, and do not include South Africa specifically are excluded:
3. All study designs were eligible, including modelling studies, narrative and systematic reviews, case studies, case series and qualitative research.
4. Articles to cover health in its broadest sense, including social determinants of health and nutrition

Exclusion criteria:

5. Articles not in English articles
6. Articles that only cover animals or plants
7. Articles on climate change adaptation or mitigation

1) Search strategy (Stage 1)

Two databases were searched: Medline (Pubmed) and Web of Science. Articles published in the past 10 years in Pubmed were included and in the last 5 years in Web of Science. The Web of Science search was restricted to the last 5 years as the search engine for that database is less sensitive than Pubmed, and thus longer time periods result in large number of 'hits', making the review not feasible.

Pubmed will be searched using a combination of free text terms and Mesh codes.

The Pubmed search strategy consists of:

((((("South Africa"[MeSH]) OR ("South Africa"[Title/Abstract]) OR ("Southern Africa*"[Title/Abstract]))) AND "last 10 years"[PDat])) AND (((“global warming”[Title/Abstract] OR “global warming”[MESH] OR climatic*[Title/Abstract] OR “climate change"[Title/Abstract] OR “climate change”[MESH] OR “Desert Climate”[mesh] OR “El Nino-Southern Oscillation”[Mesh] OR Microclimate[mesh] OR “Tropical Climate”[mesh])) AND "last 10 years"[PDat]) Sort by: PublicationDate Filters: published in the last 10 years.

The Web of Science search strategy is:

TS=(Global warming) OR TS=(climate change*) AND ((TS="South Africa" OR CU="South Africa" OR TS="South African")) AND (SU=(Environmental Sciences & Ecology) OR WC=(Medicine, Research & Experimental) OR SU=(Tropical Medicine) OR SU=(Public, Environmental & Occupational Health) OR SU=(Health Care Sciences & Services) OR SU=(Infectious Diseases) OR SU=(Research & Experimental Medicine) OR SU=(General & Internal Medicine) OR SU=(Psychiatry) OR SU=(Pediatrics) OR SU=(Green & Sustainable Science & echnology)) NOT TS=wildlife OR TS=fish OR TS=flora OR TS=plant* OR TS=conservation LANGUAGE: (English) AND DOCUMENT TYPES: (Article). Indexes=SCI-EXPANDED, SSCI, A&HCI, ESCI Timespan=Last 5 years

2) Process of screening of titles and abstracts in Stage 2: variables and instructions

This section outlines the instructions and codes used when screening of titles, and, if required, abstracts. Screening is done by a single reviewer.

Each title/abstract will be coded according to the coding system that was finalised after piloting of potential codes. Definitions are provided in the box below for each code.

Each article must be coded within only one of the categories shown in Box 1 (aside from articles coded as 'Background', which must be coded with one of the other codes. Thus, aside from the code 'Background', the categories are mutually exclusive.

Instructions for duplicate articles

Some duplicate articles may be found, the first of the duplicate articles as include/exclude or query, and then the subsequent duplicate article(s) to be coded as duplicates.

Query code: If you are unclear, code the study as a "QUERY unclear". You must note the reason you are unclear.

Eligibility criteria and coding of included articles

Use a low threshold for classifying articles as requiring a full text search (i.e. if in doubt, include the article). Thus, if it is possible that an article might be relevant, mark it as requiring full text assessment. The plan is to over-include during the screening of title/abstract, and then, if required, to exclude later on review of the full text. By 'over-including', we attempt to work on an abstract once only, and not to return to it at a later time.

Instructions for coding on title and abstract

1. Some duplicate articles may be found, please code the first of the duplicate articles as include/exclude or query, and then code the subsequent duplicate article(s) as duplicate.

2. If the study does not meet all the inclusion criteria then EXCLUDE it. For excluded articles, mark only one code. Mark the highest option, e.g. if an article describes a study among animals (not human) and is in Spanish, then mark "Not human" as "Not human" is higher on the list than "Language".

2.1 Language Only studies in English are included

2.2 Population not human. Studies on climate change and plants or animals are thus excluded. Interactions between animals or plants with humans are included, such as zoonotic infections, even if the study does not involve humans *per se*. Also studies that examine nutrition are included, such as those examining the effects of changes in crops on human nutrition.

2.3 Study does not cover health. The study has to cover health in its broadest sense, which includes the social determinants of health and nutrition, for example.

3. If an article fulfils the inclusion criteria (based on the title/abstract), then tick INCLUDE. We mark studies that cover climate change adaptation as include in the stage of screening on title and abstract. This is done as we wish to screen the full text of the article to ascertain that it does not contain text on impact or relevant references. That decision can only be made on review of the full text. To be marked as screen positive on title and abstract, the study MUST describe the impact of climate change on South Africa, or adaptation to climate change. All study designs are eligible, including modelling studies, narrative and

systematic reviews, case studies, case series and qualitative research. Also, include studies of costing of climate impact or adaptation. An example of a study to be included is a study on the impact of climate change in South Africa on health of residents in informal settlements and how they have changed the housing type in response to that.

Studies have to be set in South Africa, and may be part of multi-country studies, such as those involving Southern African countries. Studies that address Southern Africa as a broad topic, and do not include South Africa specifically are excluded: South Africa must be directly addressed in a paper for it to be included.

The ‘unit’ that is studied varies considerably. Studies to be included in the review may be provided directly to individuals or groups of people; indirectly to health or other kinds of staff who then deliver an intervention; to houses, health facilities or other types of structures, among other possibilities.

Mark articles as ‘Query’ if at all uncertain.

Variables to extract in screening of titles and abstracts (Stage 2)

1. DUPLICATE

2. **EXCLUDE** on title and/or abstract, and why excluded (hierarchy approach: mark only highest applicable item on list):

Not human

An excluded language

Not South Africa

Not climate change

Not on health

Climate change mitigation (not impact or adaptation)

3) Screening of Full Text articles in Stage 3

Here, we screen the full text of all articles coded during screening of Titles/Abstract as: Include or Query. In this stage, the full text articles are checked to ensure that the codes applied when the titles and abstracts were screened are correct. Screening is done by a single reviewer.

Please confirm that the PDF that was uploaded is the same as the abstract, some errors in uploading may occur. Delete the PDF if it is the incorrect one.

Perform Full Text Screening, by checking the article is eligible, and reclassify the codes applied during screening of the Title/Abstract, if required.

Note that some articles that were included on screening of title and abstract will be excluded on review of full text. If the full text article does not meet the inclusion criteria then EXCLUDE it. For excluded articles, mark only one code. The exclude category uses a hierarchy approach, whereby the reviewer must mark only the exclusion criteria highest on the list that applies to the study, e.g. if an article describes a study among animals (not human) and is in Spanish, then mark "Not human" as "Not human" is higher on the list than "Language".

Each article must be coded within only one of the following categories: duplicate; exclude (only one exclude category, the highest applicable category); duplicate, include (mutually exclusive categories, or query.

When screening full text articles, exclude those that only cover Climate Change adaptation.

Codes for screening of full text articles are:

1) Exclude code from one of the exclude categories used at the screening of title/abstract stage. Instructions for each code are identical to those used in Stage 2 (see above)

2) Exclude articles only on climate change adaptation
3) Include climate change impact (articles that fulfil the eligibility criteria for the review)

4) Extraction of data on Full Text articles (Stage 4)

We will extract data from included articles. We will not assess the quality of the research, as there are multiple study designs and we are not doing a full extraction of study outcomes.

Data extraction will be done by a single reviewer. Extraction is based on the abstract and full text of articles. All included articles are to be extracted, regardless of study design.

Additional references from studies done outside of South Africa may be used if relevant and not evidence exists on the topic from South African studies.

Variables to be extracted were piloted and then finalised. Data will be extracted into a Microsoft Excel document by a single reviewer.

Variables to be extracted are defined here. Mark N/A (not applicable) if a field does not apply.

Variables to be extracted from full text of all articles included after screening of full text.

1. Country(ies) (tick all that apply) where research conducted.
2. Part of South Africa where research is conducted
3. Study design. Coded as: Randomised controlled trial (RCT); Modelling; Effectiveness evaluation including process evaluation (not RCT); Qualitative design; Formative non-intervention research; case study or series; Other (details); Unclear
4. Study population unit: individual, family, school, occupational setting, other

The kind of data extracted during writing of the paper will vary by topic, type of article and setting. The data presented in each paper will be used to draft the text of the paper and from the basis for the findings reported in the paper. Each included article will be referenced after the text which was drawn from that article. Articles that cover similar topics will be grouped together to provide a summary of the findings from studies on the topic.

© 2018 by the authors. Submitted for possible open access publication under the terms and conditions of the Creative Commons Attribution (CC BY) license (http://creativecommons.org/licenses/by/4.0/).
